# Amphetamine use as a predictor of cardiovascular and cerebrovascular mortality and morbidity: a longitudinal cohort study of criminal justice clients

**DOI:** 10.3389/fcvm.2025.1378833

**Published:** 2025-01-24

**Authors:** Ada Åhman, Jonas Berge, Anders Håkansson

**Affiliations:** Department of Clinical Sciences Lund, Division of Psychiatry, Faculty of Medicine, Lund University, Lund, Sweden

**Keywords:** amphetamine, cardiovascular, cerebrovascular, mortality, morbidity, cohort study, criminal justice clients, PWID

## Abstract

**Introduction:**

Amphetamine use is an increasing problem, and studies suggest a connection between amphetamine use and cardiovascular and cerebrovascular pathology. However, few long-term studies examine amphetamine users' risk of cardiovascular and cerebrovascular pathology, in comparison to users of other drugs. In addition, in a criminal justice system, illicit drug use and psychiatric comorbidity is common, whereas structured treatment and follow-up is uncommon, and stimulant use is common in this setting. The aim of this study was to investigate the risk of cardiovascular and cerebrovascular morbidity and mortality for intravenous drug users with different drugs as the primary drug, using data from the criminal justice system.

**Methods:**

A cohort of injecting substance users (*N* = 2,422) was identified in the Swedish criminal justice system through interviews with the Addiction Severity Index (ASI) between January 2001 and August 2006. Data on age, sex, self-reported injection drug, tobacco use, and time in prison or custody were retrieved from the ASI database. The clients were followed in national registers up to 2014 with respect to cardiovascular and cerebrovascular morbidity and mortality. Potential predictors of cardiovascular and cerebrovascular events were investigated.

**Result:**

Self-reported main drug was amphetamine in 51.5% (*n* = 1,247), polysubstance use in 33% (*n* = 799), and heroin in 15.5% (*n* = 376) of the cohort. Total observational time for the entire cohort was 23,911 person-years [median 10.3 years (IQR 9.3–11.2 years)]. The highest incidence rates of both cardiovascular and cerebrovascular events were found among amphetamine users. Bivariate analyses showed a significantly higher percentage of cardiovascular events in amphetamine users compared to other substance users (*p* < 0.044). Amphetamine was not significantly associated with cardiovascular or cerebrovascular events, compared to the main drug heroin or polysubstance use.

**Conclusion:**

In this study on substance-using criminal justice clients, while the highest incidence rates of both cardiovascular and cerebrovascular events were found among amphetamine-using individuals, the study did not provide evidence of an independent association. The study highlights the need to take co-factors into account, such as comorbidities and socio-economic factors. More studies are needed to distinguish substance-specific pathology from the impact of other unhealthy lifestyle factors among substance-using individuals.

## 1 Introduction

Amphetamines play a substantial role on the drug scene all over the world with 36 million past-year users in 2021 and an increasing use in most countries over the past decade (1). Amphetamine is the most common drug among Swedish injecting drug users (2) and is the second most common drug in Swedish seizure statistics, after cannabis (3).

Amphetamines have a stimulating effect on the central nervous system and induce an increase of the endogenous catecholamines dopamine and noradrenaline (4, 5). The catecholamines stimulate peripheral alpha- and beta-adrenergic receptors, causing an instant rise in blood pressure and pulse rate (4, 5). Amphetamine use has been shown to be associated with psychological harms such as psychosis, depression, anxiety, and violent behaviours (5), and intoxications including stimulants also have been hypothesized to play a role in suicides committed through poisoning (6). A variety of physical harms has also been described, such as seizures and hyperthermia. Intravenous drug use is itself linked to several complications, amongst others, an increased risk of blood-borne virus infections. Two of the most serious physical consequences of amphetamine use are cardiovascular disease (CVD) and cerebrovascular disease (CBD) (5).

Older studies in the field have shown that amphetamine can cause acute myocardial infarction (7–10). A review article from 2007 was also able to demonstrate that methamphetamine users ran a higher risk of being affected by all types of cardiovascular pathology (11). Several later studies based on autopsies have confirmed an association between amphetamine and various cardiovascular pathology, including coronary artery atherosclerosis and hypertension (12–14). An association to cardiomyopathy has additionally been shown (13, 15, 16). Studies on mortality and causes of death among amphetamine users also show that CVD is the most common natural cause of death (17–19). Amphetamine users are also more likely to die of CVD compared to the general population (17, 18, 20).

Furthermore, there is evidence supporting a connection to cerebrovascular pathology. Kaye and colleagues (12) investigated methamphetamine-related fatalities in Australia during 2000–2005, and found cerebrovascular pathology—most frequently cerebral haemorrhage and hypoxia—present in 20% of the cases. Several other studies (19, 21, 22), including a review from 2017 (23), support an association between amphetamine use and hemorrhagic stroke. A systematic review from 2018 (24) also found some evidence that prescribed amphetamine-type stimulants increased the risk for stroke, although the review was based on a limited number of studies.

The potential association between stimulants and cardiovascular and cerebrovascular disease is, however, complex, especially due to the fact that legal medication of certain disorders also involve central stimulants such as amphetamines and amphetamine analogues. In the increasing population of patients treated with stimulants for ADHD, cardiovascular risk has been discussed and in one study not demonstrated (25), while another recent study demonstrating that the risk of such pathology increases with increasing duration of stimulant medication (26).

Specifically, in criminal justice settings, drug use, drug use disorders, and mental health-related comorbidities, are common (27). Also, it can be assumed that treatment uptake and long-term follow-up in criminal justice clients are challenging. In this specific setting, we also know that stimulants, including amphetamine, are among the most common drugs reported (17, 28).

While there is strong evidence supporting the association between amphetamine use and cardiovascular and cerebrovascular pathology there is still a lack of long-term studies which examine amphetamine users' risk of CVD and CBD, in comparison to drug users who report other drugs than amphetamine as their main drug. The aim of this study was thus to investigate the risk of CVD- or CBD-related morbidity and mortality for intravenous drug users with different drugs as the primary drug, using data from the criminal justice system. The working hypothesis was that amphetamine users differ from those who primarily use other drugs by having a higher risk of cardiovascular and cerebrovascular pathology.

## 2 Materials and methods

### 2.1 Study population

This prospective follow-up study includes subjects from a database of criminal justice clients with substance use problems. The subjects were interviewed with a standardized and structured interview instrument, the Addiction Severity Index (ASI) (29). The ASI assessment is used on people with a known or suspected misuse of drugs or alcohol, with the aim to evaluate alcohol- and drug-related problems and transferring the clients to appropriate treatment units within the prison and probation system (28, 30). The interview examines different areas such as substance use, including alcohol and tobacco use, medical and psychiatric illness history, as well as social and legal problems (29, 31).

The ASI is a well-documented instrument, developed by researchers in The United States in the seventies and now used globally (29). It was first introduced in the Swedish Prison and Probation Service in 2001 and has been in increasing use since then (31). The present ASI data used in this study has been the subject of several other longitudinal studies (28, 30, 32–34). The ASI version used in this setting, ASI-X (35), is a developed version of the European standard version of the instrument, EuropASI (36).

The interviews were performed in the Swedish Prison and Probation Service between January 2001 and August 2006. The ASI is voluntary, and approximately 6% of the clients addressed for an ASI interview in this data set refused to participate (33). Only Swedish-speaking clients were examined with the ASI. Fifty individuals were interviewed before intake to the criminal justice facility with a median of 10 days before intake. The remaining 2,372 clients had a mean number of days between intake to the time when the interview was performed of 55 days (median 27 days; 98% were interviewed within the first year) (32). A total of 2,608 participants (82%) were interviewed in custody or prison [median time of incarceration 4.8 months (range 0–166.5 months)].

The inclusion criteria for this study were self-reported regular injection use with amphetamine, heroin, or polysubstance use as the main problem. Regular injection use was defined as any injection drug use for at least six months before admission to the criminal justice facility. The main drug was defined as the substance that the interviewed subject reported as “the major problem” (37). Polysubstance use was defined as the reporting that the major problem was two or more different drugs, again during at least six months prior to admission, of which at least one drug was an illicit substance (and again including the fulfilment of the injection drug use criterion of the study).

**Figure 1:**
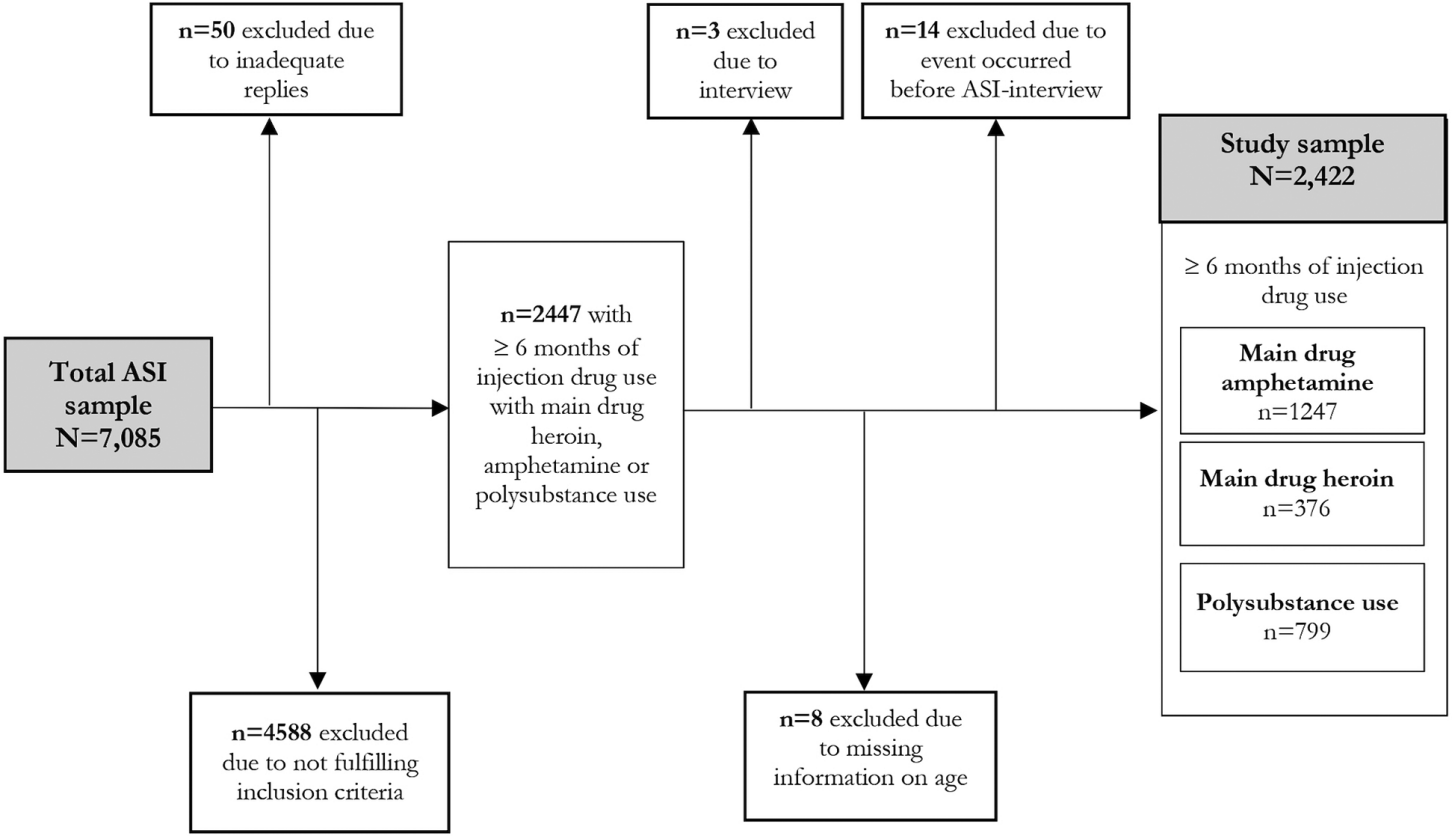
Flow-chart describing inclusion in and exclusion from the study.

A total of 7,085 individuals were interviewed during the study period. Fifty subjects were excluded due to misinterpretation of the questions, inability to understand, refusing the interview, inability to conduct the interview, or interrupted the interview. A total of 4,588 individuals were excluded due to not fulfilling the inclusion criteria of at least 6 months of injection use with predominant (main drug) use of heroin, amphetamine, or polysubstance use. The cohort then consisted of 2,447 clients. For three subjects the interview instrument used was the Adolescent Drug Abuse Diagnosis (ADAD) (38) and not a full ASI interview, and these clients were excluded. Eight individuals lacked information regarding age, these were also excluded. Lastly, 14 individuals had an event occurring before the ASI-interview was performed, and these were excluded. The study was finally based on 2,422 clients (Figure 1).

### 2.2 Variables and outcome measures

The independent variables were retrieved from the ASI database. The main variables were self-reported injection drug (predominant use of amphetamine, heroin, or polysubstance use). Amphetamine was set as the reference variable. The control variables were sex (female, male), age, tobacco use (current tobacco use or not), whether the interview took place in incarceration (custody/prison vs. not), and time in prison or custody.

Follow-up data on cardiovascular and cerebrovascular outcomes were collected from two registers: The National Patient Register (NPR) and The Causes of Death Register (CDR), held by The Swedish National Board of Health and Welfare. The NPR provides the primary and secondary diagnoses for hospital-based patients as well as patients in the specialist out care. The diagnoses are set by physicians according to the International Classification of Diseases 10 (ICD-10). The register covers 99% of all somatic and psychiatric hospital discharges and 80% of all specialized outpatient care, primary health care is not included (39). The lower coverage of the outpatient care is probably due to missing data from private caregivers and some psychiatric outpatient treatment units (40).

Underlying causes of death, and death dates, were collected from the CDR. The causes of death in the register are stated according to ICD-10 and are based on death certificates set by physicians. Deaths amongst people with known or suspected drug or alcohol use disorders, shall according to Swedish legislation, routinely be investigated through forensic autopsy, and the cause of death is set by the physician performing the examination. The CDR covers more than 99% of deaths occurring in Sweden and includes all deceased individuals that at the time of death were registered in The Swedish Population Register, between the years 1961 and 2011. From 2012 the register also includes those not registered in the Swedish Population Register (41).

The outcome variables were: (1) time to the first CVD event, and (2) time to the first CBD event. An event was defined as the first occurrence of the diagnoses ICD-codes I20–I25 (CVD events) or the diagnoses ICD-codes I60–I69 (CBD events) in the NPR *or* the CDR (Table 1). Both primary and secondary diagnoses were considered in the NPR as well as the underlying causes of death in the CDR.

**Table 1:** ICD-codes included in the two outcome variables CVD event and CBD event.

CVD event ICD 10-codes I20–I25	CBD event ICD 10-codes I60–I69
I20 angina pectoris	I60 subarachnoid hemorrhage
I21 acute myocardial infarction	I61 intracerebral hemorrhage
I22 subsequent myocardial infarction	I62 other nontraumatic intracranial hemorrhage
I23 certain current complications following myocardial infarction	I63 cerebral infarction
I24 other acute ischemic heart diseases	I64 stroke, not specified as hemorrhage or infarction
I25 chronic ischemic heart diseases	I65 occlusion and stenosis of precerebral arteries, not resulting in cerebral infarction
	I66 occlusion and stenosis of cerebral arteries, not resulting in cerebral infarction
	I67 other cerebrovascular diseases
	I68 cerebrovascular disorders in diseases classified elsewhere
	I69 sequele of cerebrovascular disease

CVD, cardiovascular disease; CBD, cerebrovascular disease.

The Prison and Probation Service and the Swedish National Board of Health and Welfare linked the data in the ASI database with the diagnosis and causes of death data from 2001 to 2014 through national identification numbers. The endpoint of the observational time was either cardiovascular or cerebrovascular diagnosis or death, or 31 December 2014, whichever occurred first.

### 2.3 Statistical analysis

Initially, bivariate analyses were performed. Dichotomous variables are presented as absolute and relative frequency and comparisons between groups were calculated with Fisher's Exact test. Differences in median age were assessed using the Mann Whitney *U* test. We used extended Cox regression models to study time to the first CVD and CBD event during the observational time. As noted earlier, an event is defined as either morbidity or mortality resulting from CVD or CBD, respectively. The presented hazard ratios (HR) from the Cox regression therefore reflect both morbidity and mortality associated with CVD and CBD. Age of the study participants was used as the time scale, as it's considered preferable to use time-on-study with age as a covariate in observational studies when the influence of age is not of primary interest (42). Incarceration (including time in custody) was set as a time-varying variable due to the fact that the follow-up data includes both time in prison and the time after release from prison, until the first occurring event. In the unadjusted model we tested every variable separately and in the adjusted model all variables were controlled for one another as they were entered simultaneously into the model.

We used the Huber Sandwich Estimator to obtain robust estimates of the standard errors in order to account for the multiple observations of some study participants. Variance inflation factors were calculated and found to be below 1.1 for every variable studied.

In order to obtain estimates for all pairwise comparisons of main drug in the Cox regression models, we performed a contrasts analysis using the *multcomp* package in R, with *p* values being adjusted using the single-step method (43). Statistical analyses were performed in R.3.3.2 (44) and in IBM Statistical Package for Social Sciences (SPSS) versions 25 and 29.

### 2.4 Ethics

Due to the study design and following the approval by the Lund Regional Ethics Board (file number 2014/478), active informed consent was not obtained. The ASI interviews were conducted routinely as part of the efforts of the criminal justice service. The ASI material did not contain any personal identifiers such as names or social security numbers when accessed by the researchers. Both our internal assessment and the evaluation conducted by the Ethics Review Board indicated that the research entailed a minimal likelihood of harm to the research subjects. Consequently, it was determined that written approval was deemed not necessary in retrospect. However, the planned study was announced in a free-of-charge newspaper in the three major cities in Sweden (Stockholm, Gothenburg, and Malmö), to give the participants an opportunity to decline contributing to the study. No individuals chose to opt-out.

## 3 Results

### 3.1 Population characteristics

The studied cohort consisted of 2,422 subjects, 339 were women {14%, median age 36 [interquartile range (IQR) 29–43 years]}, 2,083 were men [86%, median age 36 (IQR 28–43 years)] (Table 2). Self-reported predominant (main drug) use in the selected cohort was amphetamine in 51.5% (*n* = 1,247), polysubstance use in 33% (*n* = 799) and heroin in 15.5% (*n* = 376).

**Table 2:** Baseline characteristics among 2,422 criminal justice clients who reported injection drug use with a predominant (main drug) use of either amphetamine, heroin, or polysubstance use.

Variables	Outcome, % (*n*)						
	total sample *n* = 2,422	CVD event *n* = 57	No CVD event *n* = 2,365	*p*-value*	CBD event *n* = 41	No CBD event *n* = 2,381	*p*-value*
Median age, years (IQR)	36.0 (28–43)	44.0 (39–50)	36.0 (28–43)	**<0**.**001**	45.0 (38–50)	36.0 (28–43)	**<0**.**001**
Female sex	14.0 (339)	0.6 (2)	99.4 (337)	**0**.**019**	1.5 (5)	98.5 (334)	1.000
Male sex	86.0 (2,083)	2.6 (55)	97.4 (2,028)		1.7 (36)	98.3 (2,047)	
Tobacco use	94.6 (2,291)	2.3 (53)	97.7 (2,238)	0.547	1.6 (36)	98.4 (2,255)	0.067
No tobacco use	5.4 (131)	3.1 (4)	96.9 (127)		3.8 (5)	96.2 (126)	
Prison or custody	86.8 (2,102)	2.4 (50)	97.6 (2,052)	1.000	1.7 (35)	98.3 (2,067)	0.815
No prison or custody	13.2 (320)	2.2 (7)	97.8 (313)		1.9 (6)	98.1 (314)	
Main drug AMP	51.5 (1,247)	3.0 (37)	97.0 (1,210)	**0**.**044**	2.1 (26)	97.9 (1,221)	0.156
Other main drug	48.5 (1,175)	1.7 (20)	98.3 (1,155)		1.3 (15)	98.7 (1,160)	
Main drug heroin	15.5 (376)	1.1 (4)	98.9 (372)	0.093	0.5 (2)	99.5 (374)	0.078
Other main drug	84.5 (2,046)	2.6 (53)	97.4 (1,993)		1.9 (39)	98.1 (2,007)	
Polysubstance use	33.0 (799)	2.0 (16)	98.0 (783)	0.478	1.6 (13)	98.4 (786)	1.000
Other main drug	67.0 (1,623)	2.5 (41)	97.5 (1,582)		1.7 (28)	98.3 (1,595)	

Data retrieved from addiction severity index interview. Bold *p* values are significant.

CVD, cardiovascular disease; CBD, cerebrovascular disease; IQR, interquartile range; AMP, amphetamine.

* Fisher's exact test used for all categorical variables. Mann-Whitney *U* Test used for comparisons of median age between the event vs. no event groups.

Total observational time for the entire cohort was 23,911 person-years [median 10.3 years (IQR 9.3–11.2 years)]. Total observational time for the outcome variable CVD event was 23,679 person-years [median 10.3 years (IQR 9.2–11.2 years)] and 23,767 person-years [median 10.3 years (IQR 9.2–11.2 years)] for CBD event.

### 3.2 Bivariate analyses

Bivariate analyses showed a significantly higher percentage of predominant amphetamine users experiencing a CVD event compared to other substance users, 3% compared to 1.7% (*p* < 0.044) (Table 2). There was a significantly lower proportion women experiencing a CVD event, 0.6%, compared to the proportion men experiencing a CVD event, 2.6% (*p* = 0.019). The median age was significantly higher amongst those who experienced an event (CVD or CBD) than those who did not experience an event, 44 (IQR 39–50) and 45 (IQR 38–50) years respectively, compared to 36 years (IQR 28–43) (*p* < 0.001).

There were no significant differences in proportions of CVD or CBD events between tobacco users and non-users and individuals serving time in prison or custody and those who did not (Table 2).

### 3.3 Incidence of first episode of CVD and CBD event

The total number of CVD events was 57, of those were 9 events CVD-related deaths. The total number of CBD events was 41 and 9 events of those were CBD-related deaths. Person-time incidence rate of first episode of CVD event was 2.41 per 1,000 person-years for the entire study population. Person-time incidence rate of first episode of CBD events was 1.73 per 1,000 person-years for the total sample. The highest incidence rates of both CVD and CBD events were found among subjects reporting amphetamine as predominant (main drug) use (2.99 and 2.09 respectively).

### 3.4 Predictors of CVD and CBD events

In the unadjusted model, female sex was found to be associated with lower risk of CVD event (HR 0.21 [95% CI 0.05–0.87], *p* = 0.032 (Table 3). No other variable was significantly associated with CVD or CBD event. In the adjusted analysis, none of the outcome variables CVD or CBD events were significantly associated with any of the predominant (main) drugs, neither in the comparison of amphetamine with heroin or polysubstance drug use, nor in the comparison between heroin and polysubstance use. Female sex was significantly associated with lower risk of a CVD event [aHR 0.21 (95% CI 0.05–0.87), *p* = 0.032].

**Table 3:** Variables potentially associated with first CVD and CBD events during the observational time. Cox regression model with amphetamine as reference category.

Outcome	CVD event			CBD event		
	uHR (95% CI)	aHR (95% CI)	*p*-value*	uHR (95% CI)	aHR (95% CI)	*p*-value*
Amphetamine vs. heroin	1.43 (0.52–3.93)	1.38 (0.5–3.79)	0.8	2.01 (0.45–8.97)	2.04 (0.46–9.1)	0.604
Amphetamine vs. polysubstance	0.99 (0.55–1.8)	1.03 (0.57–1.86)	0.994	0.86 (0.43–1.72)	0.87 (0.44–1.74)	0.913
Heroin vs. polysubstance	0.69 (0.23–2.04)	0.75 (0.25–2.2)	0.852	0.43 (0.1–1.93)	0.43 (0.1–1.9)	0.489
Prison or custody	1.15 (0.49–2.69)	1.13 (0.48–2.64)	0.785	0.24 (0.03–1.74)	0.23 (0.03–1.71)	0.155
Female sex	0.21 (0.05–0.87)	**0.21 (0.05–0.87)**	**0**.**032**	0.82 (0.32–2.10)	0.81 (0.31–2.07)	0.653
Tobacco use	0.79 (0.28–2.18)	0.78 (0.28–2.16)	0.621	0.45 (0.17–1.15)	0.45 (0.18–1.17)	0.076

Time measured from baseline interview to first CVD and CBD event (diagnosis or death), respectively. *N*  =  2,422. Number of CVD events = 57, number of CBD events = 41. Bold *p* values are significant.

CVD, cardiovascular disease; CBD, cerebrovascular disease; uHR, unadjusted HR; aHR, adjusted HR.

* *p* values from the adjusted analysis.

## 4 Discussion

This is one of few studies investigating cardiovascular and cerebrovascular morbidity and mortality among individuals predominantly using amphetamine, in comparison to other substance users. The study has a relatively long follow up of median 10.3 years (IQR 9.3–11.2 years) and contains detailed baseline data, a feature frequently absent in longer follow-up studies of mortality and morbidity in criminal justice clients with severe substance use problems.

In the bivariate analyses we found a significantly higher percentage of CVD events in predominant users of amphetamine compared to other substance users (*p* < 0.044) (Table 2). This is consistent with the prevailing evidence showing an association between amphetamine use and various cardiovascular pathologies (11, 13). It is also consistent with the findings of Turner et al. (45) from 2018, indicating that deaths linked to stimulant use were more likely to involve cardiovascular causes than deaths linked to opioids.

The highest incidence rates of both CVD and CBD events were also found among subjects reporting predominant (main drug) use of amphetamine (2.99 and 2.09 respectively) (Table 4). Amphetamines are exogenous catecholamines, with distinctive effects such as an instant increase of heart rate and blood pressure (5). This, combined with an accelerated development of atherosclerosis (11, 12), could contribute to a higher incidence of cardiac pathology among the amphetamine users.

**Table 4:** Incidence of first CVD and CBD event (diagnosis or death), expressed as person-time incidence (number of events per 1,000 person-years) during the observational time 2001–2014.

Outcome	Study participants (*n*)	Total CVD events (*n*)	Total CBD events (*n*)	CVD events (*n* events per 1,000 person-years)	CBD events (*n* events per 1,000 person-years)
Total sample	2,422	57	41	2.41	1.73
Main drug amphetamine	1,247	37	26	2.99	2.09
Main drug heroin	376	4	2	1.14	0.57
Polysubstance use	799	16	13	2.05	1.66

Data retrieved from the national patient register and the causes of death register.

CVD, cardiovascular disease; CBD, cerebrovascular disease.

In the extended Cox regression analysis, no one of the variables describing predominant (main drug) use of heroin or polysubstance use were significantly associated with the outcome variables CVD or CBD event, compared to amphetamine. The only significant association of the control variables was female sex, which was associated with lower risk of a CVD event, in line with previous evidence (46). This result could be due to the study sample not being large enough to identify a difference between the groups. Consistent with previous literature (47), we estimated that approximately 10 events were needed for each included predictor variable. However, this estimation was based on the literature, and it is possible that a larger number of study participants was required in this case. It is also possible that a wider selection of CVD, for example including cardiomyopathies and hypertension, would generate a different result. This was not feasible in our case since chronic diagnoses mainly are handled in the primary care which unfortunately not is covered by the NPR. Future studies on this subject should however consider including a wide range of CVD since amphetamine has been associated with a wide range of cardiovascular morbidity.

Furthermore, the difference between the groups in this specific cohort of substance users may be relatively small. During the follow up period of a median 10.3 years, the number of CVD events was 57 and the number of CBD events was 41, lower than we had anticipated since an event was defined as death *or* diagnosis, which ever occurred first. Most deaths of released prisoners in general (not exclusively substance users) are due to non-natural causes, particularly homicide, suicide, and drug overdose (48) and most deaths in prisons are due to suicide (49). The predominantly amphetamine-using group in this study may, to a greater extent than amphetamine users in general, die of external causes before they receive a CVD or CBD related diagnosis or die a CVD or CBD related death. Thus, it is conceivable that the impact of amphetamine use in this setting are outweighed by the impact of the lifestyle and personality traits associated with criminal justice clients. The amphetamine group would then have more similarities to the other substance using groups—making any potential group difference smaller.

Moreover, some studies indicate that incarceration might function as a health-promoting factor, especially when it comes to natural diseases (50, 51), possibly by providing better access to health care than in the community (52) but potentially also by reducing the drug use. This could mean that any group differences in cardiovascular and cerebrovascular morbidity and mortality between different substance users in the prison environment become less apparent.

The present study sheds light on the need to address a number of co-factors in the analysis of whether and how stimulant who predicts CVD or CBD. For example, and not unexpectedly, people with these pathologies in the present study were older than those without CVD or CBD, such that age—for obvious reasons—needs to be taken into account in this context as in other cardiovascular or cerebrovascular disease. Likewise, sex appeared to have a role in this association as the diseases studied here were markedly more common among men in the study. This bivariate finding—in the present type of study—demonstrates the need to further take sex into account when studying CVD and CBD also in this context of drug use and in the narrower group of criminal justice clients; cardio-vascular risk differs between sexes and although a number of cardio-vascular risk factors may in absolute numbers be more common in men, although their relative impact on cardio-vascular risk may not be the same in women and men (53), adding to the complexity of studies like this one. Thus, again, this finding highlights the need to address a multitude of health-related and socio-demographic risk factors when analyzing the role of illicit drug use in the prediction of disease.

Hence, this study on substance using criminal justice clients did not provide enough evidence to reject the null hypothesis, i.e., that there is no difference between substance-using groups regarding cardiovascular and cerebrovascular mortality and morbidity. More studies are needed, examining amphetamine-using groups outside the criminal justice system, and including a large number of study participants and possibly a wide range of CVD.

### 4.1 Limitations

The ASI database used in this study is based on self-reports which implies a risk of recall bias. Self-reports in this setting have, however, been shown to have high reliability (54, 55). Furthermore, our knowledge is limited to the self-reported main drug at baseline, and the study participants may have changed their main drug during the course of the study. Moreover, this study includes injecting substance users in the criminal justice system and the result should not be generalized to substance users with other routes of administration. Also, this study was performed in a criminal justice setting and the result should therefore not be generalized to other settings of substance users. Importantly, the ASI database used here has been shown to include a certain overrepresentation of women and clients sentenced with the types of crimes which are more typically associated with the drug use itself (56). Also, the present sample includes a high proportion of clients with predominant use of amphetamine. However, although data from the full general population are lacking and cannot describe the full picture of injection drug use in the whole population, our predominance of clients using amphetamine is consistent with other more clinical samples from the present setting, such as in data from needle exchange programs (2). In the latter study, past-12-month drug use was amphetamine in 43% of cases (compared to 37% for heroin), and in a needle exchange sample from a different region, amphetamine was the primary drug reported by 56% of clients and heroin by 42% (18, 57). In the database used for the present study, 52% of clients in the whole criminal justice dataset had a history of amphetamine use, compared to 18% for heroin, and 30% of clients reporting a primary drug reported that to be amphetamine, compared to 9% for heroin (56, 58). Thus, the high proportion of predominant amphetamine use in the present study can be considered to reflect the clinical picture of illicit drug use in the present geographical setting.

Moreover, 20% of all specialized outpatient care as well as the primary health care is not covered by NPR (39) and is thereby not represented in the study. The IPR have an overall validity of 85%–95% when compared to inpatient records and the proportion of valid diagnoses is probably higher in patients with severe diseases in contrast to mild or more chronic diseases like hypertension and lipid disorders (39). However, in this study we mainly included acute and severe diseases (Table 1), and the validity of the diagnosis from the IPR could therefore be assumed to be high.

### 4.2 Conclusion

This study on substance-using criminal justice clients did not provide enough evidence to conclude any differences in the risk of CVD or CBD events between the substance-using groups. However, the highest incidence rates of both CVD and CBD events were found among predominantly amphetamine-using individuals. Implications of the present paper include the need to disseminate knowledge about somatic risk factors in both amphetamine users and users of other illicit drugs, including the larger risk seen in absolute numbers among amphetamine users, but also the need for clinicians and researchers to highlight co-factors which contribute to this risk increase beyond the substance itself. More studies are needed to assess whether amphetamine use is a risk factor for CVD or CBD in comparison to other drugs and distinguish substance-specific pathology from the impact of other unhealthy lifestyle factors among substance-using individuals.

## Data Availability

The datasets presented in this article are not readily available because of Swedish legislation, which prohibits public sharing of the dataset. All data utilized for this study have been obtained from the ASI database maintained by The Swedish Prison and Probation Service. Authorization to share the data is governed by both ethics approval and permission from The Swedish Prison and Probation Service. Requests to access the datasets should be directed to The Swedish Prison and Probation Service (phone: +460772280800, email: hk.fou@kriminalvarden.se).
